# Tris(1,10-phenanthroline-κ^2^ *N*,*N*′)nickel(II) dinitrate tetra­hydrate

**DOI:** 10.1107/S160053681104880X

**Published:** 2011-11-23

**Authors:** Masoumeh Tabatabaee, Nikoo Zaji, Masood Parvez

**Affiliations:** a Department of Chemistry, Yazd Branch, Islamic Azad University, Yazd, Iran; b Department of Chemistry, The University of Calgary, 2500 University Drive NW, Calgary, Alberta, Canada T2N 1N4

## Abstract

In the title complex, [Ni(C_12_H_8_N_2_)_3_](NO_3_)_2_·4H_2_O, the Ni^II^ ion is octa­hedrally coordinated by three bidentate 1,10-phenanthroline ligands, each forming a five-membered chelate ring. In the crystal, O—H⋯O and C—H⋯O hydrogen bonds are present between the complex cations, nitrate anions and water mol­ecules. O—H⋯O hydrogen bonds between the uncoord­inated water mol­ecules lead to the formation of a four-membered ring water cluster, with a planar configuration. There were an additional five grossly disordered water mol­ecules in the asymmetric unit, which were removed by the subroutine SQUEEZE; these were were excluded in the calculation of the molecular weight, *etc*. π–π stacking inter­actions between the aromatic rings are also observed [centroid–centroid distances = 3.697 (2), 3.728 (2) and 3.761 (2) Å].

## Related literature

For background information on Ni–phenanthroline complexes and related structures, see: Qiua *et al.* (2011 ▶). For water clusters, see: Rodríguez-Cuamatzi *et al.* (2004 ▶); Sharif *et al.* (2010 ▶). For FTIR spectra of phenanthroline complexes, see: Schilt & Taylor (1959 ▶). For the synthesis of 4-amino-5-methyl-2*H*-1,2,4-triazole-3(4*H*)-thione, see: Beyer & Kröger (1960 ▶).
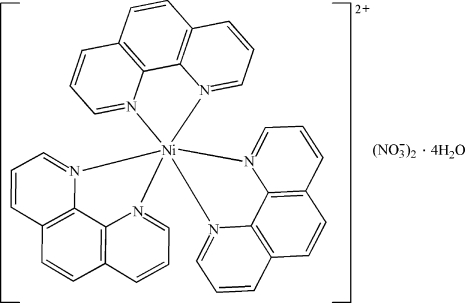


## Experimental

### 

#### Crystal data

[Ni(C_12_H_8_N_2_)_3_](NO_3_)_2_·4H_2_O
*M* *_r_* = 795.41
Triclinic, 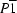

*a* = 13.0463 (6) Å
*b* = 13.1785 (5) Å
*c* = 13.4093 (4) Å
α = 82.688 (2)°
β = 72.147 (2)°
γ = 67.402 (2)°
*V* = 2025.85 (14) Å^3^
*Z* = 2
Mo *K*α radiation
μ = 0.54 mm^−1^
*T* = 173 K
0.14 × 0.12 × 0.10 mm

#### Data collection

Nonius KappaCCD diffractometer with APEXII CCD
Absorption correction: multi-scan (*SORTAV*; Blessing, 1997 ▶) *T*_min_ = 0.928, *T*_max_ = 0.948
13391 measured reflections
7100 independent reflections
5845 reflections with *I* > 2σ(*I*)
*R*_int_ = 0.037

#### Refinement

*R*[*F* ^2^ > 2σ(*F* ^2^)] = 0.057
*wR*(*F* ^2^) = 0.160
*S* = 1.08
7100 reflections
496 parameters
H-atom parameters constrained
Δρ_max_ = 0.42 e Å^−3^
Δρ_min_ = −0.39 e Å^−3^

### 

Data collection: *COLLECT* (Hooft, 1998 ▶); cell refinement: *DENZO* (Otwinowski & Minor, 1997 ▶); data reduction: *SCALEPACK*; program(s) used to solve structure: *SIR92* (Altomare *et al.*, 1994 ▶); program(s) used to refine structure: *SHELXL97* (Sheldrick, 2008 ▶); molecular graphics: *ORTEP-3* (Farrugia, 1997 ▶); software used to prepare material for publication: *SHELXL97* and *PLATON* (Spek, 2009 ▶).

## Supplementary Material

Crystal structure: contains datablock(s) global, I. DOI: 10.1107/S160053681104880X/hy2480sup1.cif

Structure factors: contains datablock(s) I. DOI: 10.1107/S160053681104880X/hy2480Isup2.hkl

Additional supplementary materials:  crystallographic information; 3D view; checkCIF report

## Figures and Tables

**Table 1:** Hydrogen-bond geometry (Å, °)

*D*—H⋯*A*	*D*—H	H⋯*A*	*D*⋯*A*	*D*—H⋯*A*
O7—H72⋯O9^i^	0.82	1.97	2.768 (5)	164
O8—H81⋯O7^i^	0.82	1.94	2.749 (5)	172
O8—H82⋯O2^ii^	0.82	2.04	2.827 (4)	164
O9—H91⋯O4	0.82	2.07	2.845 (6)	155
O9—H92⋯O6^i^	0.82	2.00	2.817 (6)	175
O10—H101⋯O8	0.82	2.01	2.822 (5)	169
O10—H102⋯O9	0.82	2.20	2.900 (6)	143
C5—H5⋯O5^iii^	0.95	2.53	3.293 (7)	138
C15—H15⋯O2^iv^	0.95	2.53	3.265 (5)	134
C25—H25⋯O7^v^	0.95	2.52	3.300 (5)	140
C32—H32⋯O4	0.95	2.46	3.165 (6)	131
C34—H34⋯O1^ii^	0.95	2.37	3.180 (5)	143

Symmetry codes: (i) 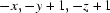; (ii) 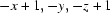; (iii) 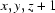; (iv) 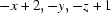; (v) 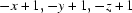.

**Fig. 1.**
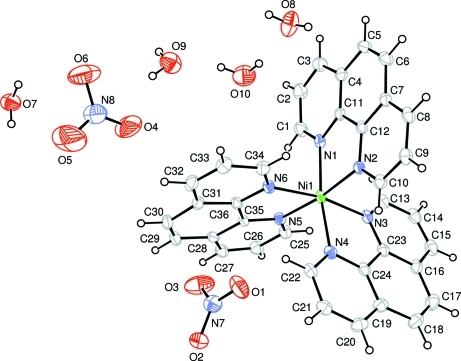
Molecular structure of the title compound. Displacement ellipsoids are drawn at the 50% probability level.

**Fig. 2.**
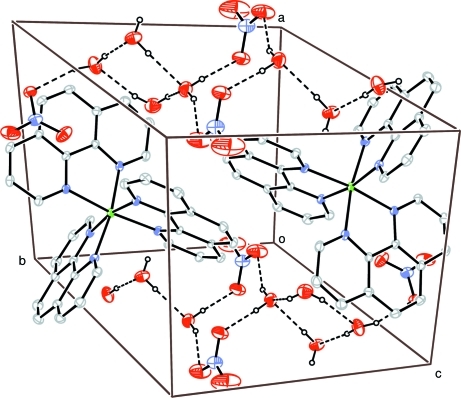
A unit cell packing of the title compound. Hydrogen bonds have been plotted with dashed lines.

**Fig. 3.**
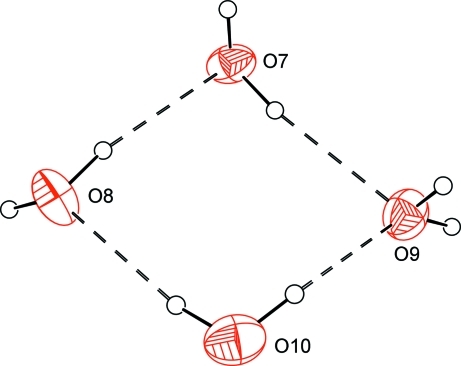
A four-membered ring water cluster formed by hydrogen bonding interactions (dashed lines).

**Fig. 4.**
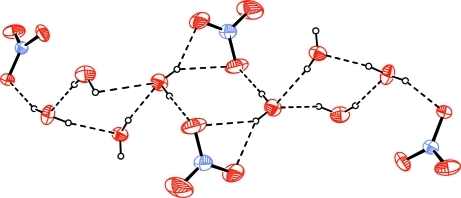
A one-dimensional chain formed by hydrogen bonds (dashed lines) between the water clusters and nitrate anions.

## supplementary crystallographic information

### Comment

Metal complexes with polypyridine-containing ligands are of great interest to researchers, and a number of reports have been devoted to this theme. Among polypyridine-containing ligands, 1,10-phenanthroline (1,10-phen) is a very important pyridine derivative, which has attracted much interest in coordination chemistry. Transition metal complexes coordinated with 1,10-phen show excellent photoelectrical capability. The application of a Ni–phenanthroline complex for detection of nucleic acid has also been reported (Qiua *et al.*, 2011). Extensive investigations of small and medium size water cluster structures (H_2_O)_n_ (n = 2–100) have been reported in recent years (Rodríguez-Cuamatzi *et al.*, 2004). Among the water clusters, the cyclic (H_2_O)_4_ is very interesting as it is a simple two-structure model for liquid water. Recently we have reported a tetrameric water cluster ring in the crystal structure of a new proton transfer system derived from pyridine-2,6-dicarboxylic acid and 2-amino-4-methylpyridine (Sharif *et al.*, 2010). Here, we present the preparation and the crystal structure of the title compound and the formation of a tetrameric water cluster with a planar configuration.

The title compound is built up of a [Ni(1,10-phen)_3_]^2+^ complex cation, two NO_3_^-^ anions and four uncoordinated water molecules (Fig. 1). In the cation, the Ni^II^ ion is octahedrally coordinated by three bidentate 1,10-phen ligands, each forming a five-membered chelate ring. The Ni—N bond distances in the cation are in accord with the values reported for complexes which contain the same ligand (Qiua *et al.*, 2011). The bond angles around the Ni^II^ ion involving *trans* pairs of donor atoms are in a range of 169.85 (10)–172.77 (11)°, while for the *cis* pairs this range is 79.72 (11)–96.51 (11)°. These values indicate a distortion from an ideal octahedral geometry. The crystal packing of the complex is dominated by numerous hydrogen bonds of the types O—H···O and C—H···O (Fig. 2, Table 1). Hydrogen bonding interactions between the uncoordinated water molecules lead to the formation of a four-membered ring water cluster with a planar configuration (Fig. 3). Moreover, two planar water cluster rings are connected *via* hydrogen bonds between the nitrate anions and water molecules (Fig. 4). There are also some π–π stacking interactions between the aromatic rings, *e.g.**Cg*1···*Cg*2^i^ = 3.697 (2), *Cg*3···*Cg*4^ii^ = 3.729 (2) and *Cg*5···*Cg*5^iii^ = 3.761 (2) Å [*Cg*1, *Cg*2, *Cg*3, *Cg*4 and *Cg*5 are the centroids of N2/C7–C10/C12 ring, C4–C7/C11/C12 ring, N4/C19–C22/C24 ring, C16–C19/C23/C24 ring and C28–C31/C35/C36 ring. Symmetry codes: (i) 1-*x*, -*y*, 2-*z*; (ii) 2-*x*, -*y*, 1-*z*; (iii) 1-*x*, 1-*y*, 1-*z*].

### Experimental

All purchased chemicals were of reagent grade and used without further purification. 4-Amino-5-methyl-2*H*-1,2,4-triazole-3(4*H*)-thione (AMTT) was prepared according to the literature procedure (Beyer & Kröger, 1960). AMTT (0.260 g, 2 mmol) and NaOH (0.080 g, 2 mmol) were dissolved in 10 ml deionized water containing 1,10-phenanthroline hydrate (0.396 g, 2 mmol). A water solution (20 ml) of Ni(NO_3_)_2_.6H_2_O (0.290 g, 1 mmol) was added to the above solution. The reaction mixture was placed in a Parr-Teflon lined stainless steel vessel. It was sealed and heated at 403 K for 8 h. Then it was gradually cooled to room temperature and kept at 277 K until pink crystals suitable for X-ray diffraction were obtained (yield: 73% based on 1,10-phenanthroline).

### Refinement

The asymmetric unit of the title compound contains nine water molecules, five of which were disordered and were therefore removed by the command *SQUEEZE* in *PLATON* (Spek, 2009). They have been excluded in the calculation of molecular weight, crystal density and absorption coefficient. Although the H atoms were located from difference Fourier maps, they were included at geometrically idealized positions with O—H = 0.82 and C—H = 0.95 Å and with *U*_iso_(H) = 1.2*U*_eq_(O,C).

### Crystal data

**Table:**

[Ni(C_12_H_8_N_2_)_3_](NO_3_)_2_·4H_2_O	*Z* = 2
*M**_r_* = 795.41	*F*(000) = 824
Triclinic, *P*1	*D*_x_ = 1.304 Mg m^−^^3^
Hall symbol: -P 1	Mo *K*α radiation, λ = 0.71073 Å
*a* = 13.0463 (6) Å	Cell parameters from 8521 reflections
*b* = 13.1785 (5) Å	θ = 1.0–27.5°
*c* = 13.4093 (4) Å	µ = 0.54 mm^−^^1^
α = 82.688 (2)°	*T* = 173 K
β = 72.147 (2)°	Prism, pink
γ = 67.402 (2)°	0.14 × 0.12 × 0.10 mm
*V* = 2025.85 (14) Å^3^	

### Data collection

**Table:**

Nonius KappaCCD diffractometer with APEXII CCD	7100 independent reflections
Radiation source: fine-focus sealed tube	5845 reflections with *I* > 2σ(*I*)
graphite	*R*_int_ = 0.037
ω and φ scans	θ_max_ = 25.0°, θ_min_ = 2.8°
Absorption correction: multi-scan (*SORTAV*; Blessing, 1997)	*h* = −15→15
*T*_min_ = 0.928, *T*_max_ = 0.948	*k* = −15→15
13391 measured reflections	*l* = −15→15

### Refinement

**Table:**

Refinement on *F*^2^	Primary atom site location: structure-invariant direct methods
Least-squares matrix: full	Secondary atom site location: difference Fourier map
*R*[*F*^2^ > 2σ(*F*^2^)] = 0.057	Hydrogen site location: inferred from neighbouring sites
*wR*(*F*^2^) = 0.160	H-atom parameters constrained
*S* = 1.08	*w* = 1/[σ^2^(*F*_o_^2^) + (0.0795*P*)^2^ + 1.9793*P*] where *P* = (*F*_o_^2^ + 2*F*_c_^2^)/3
7100 reflections	(Δ/σ)_max_ = 0.001
496 parameters	Δρ_max_ = 0.42 e Å^−^^3^
0 restraints	Δρ_min_ = −0.39 e Å^−^^3^

### Special details

**Table:**

Experimental. IR Spectra IR spectra were recorded using FTIR Spectra Bruker Tensor 27 spectrometer (KBr pellets, 4000–400 cm^-1^). TGA-DTA measurements were performed at heating rate of 10 K min^-1^ in the temperature range of 298–1273 K, under nitrogen flow of 20 ml min^-1^ on instrument Perkin Elmer Pyris Diamond Thermogravimetric/Differential Thermal Analyzer. Elemental analyses were performed using a Costech ECS 4010 CHNS analyzer.The FTIR spectrum of the crystals shows broad strong bands at the region 3000–3500 cm^-1^. In the spectra of the phenanthroline complexes strong bands are observed in three frequency regions, between 700 - 900 cm^-1^, 1125 - 1250 cm^-1^, and 1400 - 1650 cm^-1^ (Schilt & Taylor 1959). In the title complex, these bonds were observed in the regions 721–869 cm ^-1^, 1138- 1225 cm ^-1^ and 1429–1573 in the IR spectra.Thermal Analyses The thermogravimetric analysis curve for compound shows that the weight loss from at 635 K corresponds to the loss of H_2_O (experimental value 18.76% and calculated value 18.29%). The further decomposition began at 801 K and finished at 803 K indicating the complete removal of organic part of the compound (experimental value 93.34% and calculated value 91.5%).
Geometry. All esds (except the esd in the dihedral angle between two l.s. planes) are estimated using the full covariance matrix. The cell esds are taken into account individually in the estimation of esds in distances, angles and torsion angles; correlations between esds in cell parameters are only used when they are defined by crystal symmetry. An approximate (isotropic) treatment of cell esds is used for estimating esds involving l.s. planes.
Refinement. Refinement of *F*^2^ against ALL reflections. The weighted *R*-factor *wR* and goodness of fit *S* are based on *F*^2^, conventional *R*-factors *R* are based on *F*, with *F* set to zero for negative *F*^2^. The threshold expression of *F*^2^ > σ(*F*^2^) is used only for calculating *R*-factors(gt) *etc*. and is not relevant to the choice of reflections for refinement. *R*-factors based on *F*^2^ are statistically about twice as large as those based on *F*, and *R*- factors based on ALL data will be even larger.

### Fractional atomic coordinates and isotropic or equivalent isotropic displacement parameters (Å^2^)

**Table:**

	*x*	*y*	*z*	*U*_iso_*/*U*_eq_	
Ni1	0.63475 (4)	0.15916 (3)	0.69156 (3)	0.02848 (15)	
N1	0.4993 (3)	0.2563 (2)	0.8096 (2)	0.0325 (6)	
N2	0.5688 (2)	0.0406 (2)	0.77467 (19)	0.0290 (6)	
N3	0.7612 (2)	0.1153 (2)	0.77055 (19)	0.0311 (6)	
N4	0.7707 (2)	0.0408 (2)	0.5899 (2)	0.0324 (6)	
N5	0.6785 (2)	0.2890 (2)	0.6102 (2)	0.0301 (6)	
N6	0.5324 (2)	0.2015 (2)	0.5887 (2)	0.0312 (6)	
C1	0.4656 (4)	0.3632 (3)	0.8248 (3)	0.0434 (9)	
H1	0.5112	0.4022	0.7816	0.052*	
C2	0.3655 (4)	0.4211 (3)	0.9020 (3)	0.0510 (10)	
H2	0.3432	0.4980	0.9093	0.061*	
C3	0.3003 (4)	0.3658 (3)	0.9667 (3)	0.0458 (9)	
H3	0.2327	0.4039	1.0198	0.055*	
C4	0.3341 (3)	0.2526 (3)	0.9540 (2)	0.0356 (8)	
C5	0.2720 (3)	0.1873 (3)	1.0187 (3)	0.0408 (9)	
H5	0.2043	0.2209	1.0736	0.049*	
C6	0.3093 (3)	0.0789 (3)	1.0020 (3)	0.0408 (8)	
H6	0.2670	0.0374	1.0461	0.049*	
C7	0.4095 (3)	0.0241 (3)	0.9210 (2)	0.0338 (7)	
C8	0.4522 (3)	−0.0897 (3)	0.8999 (3)	0.0380 (8)	
H8	0.4134	−0.1351	0.9417	0.046*	
C9	0.5486 (3)	−0.1334 (3)	0.8200 (3)	0.0385 (8)	
H9	0.5778	−0.2097	0.8061	0.046*	
C10	0.6051 (3)	−0.0662 (3)	0.7581 (3)	0.0349 (8)	
H10	0.6720	−0.0983	0.7019	0.042*	
C11	0.4337 (3)	0.2018 (3)	0.8734 (2)	0.0305 (7)	
C12	0.4717 (3)	0.0857 (3)	0.8554 (2)	0.0298 (7)	
C13	0.7583 (3)	0.1566 (3)	0.8574 (2)	0.0359 (8)	
H13	0.6927	0.2187	0.8881	0.043*	
C14	0.8470 (4)	0.1129 (3)	0.9051 (3)	0.0439 (9)	
H14	0.8424	0.1459	0.9661	0.053*	
C15	0.9405 (4)	0.0228 (3)	0.8640 (3)	0.0449 (9)	
H15	1.0004	−0.0089	0.8974	0.054*	
C16	0.9484 (3)	−0.0236 (3)	0.7721 (3)	0.0376 (8)	
C17	1.0444 (4)	−0.1179 (3)	0.7218 (3)	0.0504 (10)	
H17	1.1066	−0.1532	0.7521	0.060*	
C18	1.0475 (3)	−0.1572 (3)	0.6320 (3)	0.0475 (9)	
H18	1.1113	−0.2203	0.6008	0.057*	
C19	0.9565 (3)	−0.1053 (3)	0.5835 (3)	0.0393 (8)	
C20	0.9564 (3)	−0.1402 (3)	0.4882 (3)	0.0440 (9)	
H20	1.0187	−0.2022	0.4532	0.053*	
C21	0.8667 (3)	−0.0848 (3)	0.4469 (3)	0.0423 (9)	
H21	0.8663	−0.1075	0.3826	0.051*	
C22	0.7751 (3)	0.0058 (3)	0.4994 (2)	0.0368 (8)	
H22	0.7134	0.0441	0.4691	0.044*	
C23	0.8561 (3)	0.0273 (3)	0.7274 (2)	0.0322 (7)	
C24	0.8607 (3)	−0.0132 (3)	0.6311 (2)	0.0326 (7)	
C25	0.7471 (3)	0.3352 (3)	0.6247 (3)	0.0359 (8)	
H25	0.7780	0.3117	0.6829	0.043*	
C26	0.7760 (3)	0.4173 (3)	0.5578 (3)	0.0399 (8)	
H26	0.8239	0.4496	0.5717	0.048*	
C27	0.7346 (3)	0.4498 (3)	0.4731 (3)	0.0396 (8)	
H27	0.7558	0.5030	0.4257	0.047*	
C28	0.6603 (3)	0.4045 (3)	0.4557 (2)	0.0335 (8)	
C29	0.6100 (3)	0.4353 (3)	0.3700 (3)	0.0407 (9)	
H29	0.6297	0.4870	0.3194	0.049*	
C30	0.5352 (3)	0.3921 (3)	0.3601 (3)	0.0412 (9)	
H30	0.5029	0.4142	0.3026	0.049*	
C31	0.5035 (3)	0.3138 (3)	0.4343 (2)	0.0340 (7)	
C32	0.4228 (3)	0.2689 (3)	0.4297 (3)	0.0422 (9)	
H32	0.3850	0.2913	0.3760	0.051*	
C33	0.3996 (3)	0.1932 (3)	0.5033 (3)	0.0431 (9)	
H33	0.3450	0.1628	0.5015	0.052*	
C34	0.4568 (3)	0.1606 (3)	0.5812 (3)	0.0376 (8)	
H34	0.4408	0.1067	0.6310	0.045*	
C35	0.6342 (3)	0.3242 (3)	0.5274 (2)	0.0278 (7)	
C36	0.5545 (3)	0.2786 (2)	0.5166 (2)	0.0286 (7)	
N7	0.7221 (3)	0.0523 (3)	0.2402 (3)	0.0523 (9)	
O1	0.6907 (3)	−0.0150 (3)	0.2998 (2)	0.0791 (11)	
O2	0.7909 (2)	0.0240 (2)	0.1500 (2)	0.0519 (7)	
O3	0.6849 (3)	0.1498 (3)	0.2673 (3)	0.0939 (13)	
N8	0.1200 (4)	0.3433 (4)	0.3285 (4)	0.0794 (12)	
O4	0.1898 (4)	0.3125 (5)	0.3753 (4)	0.1248 (19)	
O5	0.1483 (6)	0.3317 (8)	0.2355 (4)	0.198 (4)	
O6	0.0122 (4)	0.3875 (4)	0.3707 (5)	0.133 (2)	
O7	0.0438 (3)	0.7131 (3)	0.2656 (2)	0.0672 (9)	
H71	0.0963	0.6673	0.2233	0.081*	
H72	0.0004	0.6814	0.2998	0.081*	
O8	0.1078 (3)	0.2081 (3)	0.8549 (3)	0.0772 (10)	
H81	0.0596	0.2280	0.8225	0.093*	
H82	0.1271	0.1419	0.8650	0.093*	
O9	0.0897 (3)	0.3860 (3)	0.5860 (3)	0.0781 (10)	
H91	0.0999	0.3794	0.5230	0.094*	
H92	0.0559	0.4519	0.5987	0.094*	
O10	0.2177 (3)	0.3503 (3)	0.7381 (3)	0.0862 (11)	
H101	0.1943	0.3028	0.7709	0.103*	
H102	0.1700	0.3890	0.7071	0.103*	

### Atomic displacement parameters (Å^2^)

**Table:**

	*U*^11^	*U*^22^	*U*^33^	*U*^12^	*U*^13^	*U*^23^
Ni1	0.0334 (3)	0.0289 (2)	0.0266 (2)	−0.01518 (19)	−0.01072 (17)	0.00554 (16)
N1	0.0399 (17)	0.0291 (15)	0.0298 (14)	−0.0134 (13)	−0.0128 (12)	0.0056 (11)
N2	0.0303 (15)	0.0294 (14)	0.0287 (13)	−0.0124 (12)	−0.0104 (11)	0.0049 (11)
N3	0.0377 (16)	0.0354 (15)	0.0265 (13)	−0.0198 (13)	−0.0124 (12)	0.0075 (11)
N4	0.0354 (16)	0.0366 (15)	0.0306 (14)	−0.0199 (13)	−0.0095 (12)	0.0034 (11)
N5	0.0309 (15)	0.0307 (14)	0.0304 (13)	−0.0147 (12)	−0.0079 (11)	0.0031 (11)
N6	0.0328 (16)	0.0334 (15)	0.0299 (13)	−0.0153 (13)	−0.0103 (12)	0.0051 (11)
C1	0.053 (2)	0.0336 (19)	0.0410 (19)	−0.0179 (18)	−0.0103 (17)	0.0055 (15)
C2	0.064 (3)	0.036 (2)	0.046 (2)	−0.011 (2)	−0.015 (2)	−0.0028 (17)
C3	0.048 (2)	0.043 (2)	0.040 (2)	−0.0115 (19)	−0.0085 (17)	−0.0057 (16)
C4	0.0347 (19)	0.041 (2)	0.0307 (16)	−0.0105 (16)	−0.0139 (14)	0.0007 (14)
C5	0.032 (2)	0.055 (2)	0.0324 (17)	−0.0166 (18)	−0.0060 (15)	0.0022 (16)
C6	0.039 (2)	0.051 (2)	0.0377 (18)	−0.0256 (18)	−0.0092 (16)	0.0062 (16)
C7	0.0348 (19)	0.0380 (19)	0.0340 (17)	−0.0180 (16)	−0.0153 (15)	0.0094 (14)
C8	0.045 (2)	0.043 (2)	0.0382 (18)	−0.0282 (18)	−0.0181 (16)	0.0126 (15)
C9	0.051 (2)	0.0301 (18)	0.0426 (19)	−0.0200 (17)	−0.0195 (17)	0.0057 (15)
C10	0.038 (2)	0.0327 (18)	0.0378 (18)	−0.0156 (16)	−0.0137 (15)	0.0039 (14)
C11	0.0327 (18)	0.0319 (17)	0.0289 (16)	−0.0118 (15)	−0.0144 (14)	0.0070 (13)
C12	0.0307 (18)	0.0363 (18)	0.0284 (15)	−0.0167 (15)	−0.0136 (13)	0.0069 (13)
C13	0.046 (2)	0.044 (2)	0.0267 (16)	−0.0265 (17)	−0.0114 (15)	0.0066 (14)
C14	0.053 (2)	0.062 (3)	0.0330 (18)	−0.035 (2)	−0.0191 (17)	0.0083 (17)
C15	0.045 (2)	0.065 (3)	0.041 (2)	−0.034 (2)	−0.0245 (17)	0.0165 (18)
C16	0.036 (2)	0.042 (2)	0.0420 (19)	−0.0214 (17)	−0.0164 (15)	0.0137 (15)
C17	0.038 (2)	0.056 (2)	0.062 (3)	−0.020 (2)	−0.0227 (19)	0.015 (2)
C18	0.033 (2)	0.040 (2)	0.065 (3)	−0.0083 (17)	−0.0145 (18)	0.0019 (18)
C19	0.0330 (19)	0.0363 (19)	0.048 (2)	−0.0170 (16)	−0.0064 (16)	0.0028 (15)
C20	0.038 (2)	0.035 (2)	0.055 (2)	−0.0116 (17)	−0.0053 (17)	−0.0088 (17)
C21	0.049 (2)	0.043 (2)	0.0358 (18)	−0.0214 (19)	−0.0062 (17)	−0.0043 (16)
C22	0.044 (2)	0.042 (2)	0.0277 (16)	−0.0199 (17)	−0.0104 (15)	0.0029 (14)
C23	0.0328 (18)	0.0389 (19)	0.0326 (16)	−0.0232 (16)	−0.0106 (14)	0.0092 (14)
C24	0.0357 (19)	0.0366 (18)	0.0307 (16)	−0.0211 (16)	−0.0097 (14)	0.0079 (14)
C25	0.0359 (19)	0.0400 (19)	0.0383 (18)	−0.0209 (16)	−0.0126 (15)	0.0055 (15)
C26	0.039 (2)	0.038 (2)	0.045 (2)	−0.0216 (17)	−0.0085 (16)	0.0032 (16)
C27	0.040 (2)	0.0344 (19)	0.0402 (19)	−0.0176 (17)	−0.0038 (16)	0.0064 (15)
C28	0.0317 (18)	0.0293 (17)	0.0284 (16)	−0.0070 (14)	0.0010 (13)	0.0008 (13)
C29	0.049 (2)	0.0376 (19)	0.0295 (17)	−0.0140 (17)	−0.0084 (15)	0.0091 (14)
C30	0.049 (2)	0.038 (2)	0.0274 (17)	−0.0034 (17)	−0.0139 (15)	0.0023 (14)
C31	0.0328 (19)	0.0327 (18)	0.0306 (16)	−0.0026 (15)	−0.0116 (14)	−0.0027 (13)
C32	0.041 (2)	0.044 (2)	0.0418 (19)	−0.0068 (17)	−0.0212 (17)	−0.0035 (16)
C33	0.041 (2)	0.046 (2)	0.053 (2)	−0.0189 (18)	−0.0206 (17)	−0.0048 (17)
C34	0.036 (2)	0.040 (2)	0.0420 (19)	−0.0177 (16)	−0.0136 (15)	0.0016 (15)
C35	0.0294 (17)	0.0278 (16)	0.0227 (14)	−0.0092 (14)	−0.0058 (13)	0.0042 (12)
C36	0.0323 (18)	0.0266 (16)	0.0239 (15)	−0.0087 (14)	−0.0061 (13)	−0.0012 (12)
N7	0.043 (2)	0.075 (3)	0.051 (2)	−0.0298 (19)	−0.0183 (16)	−0.0039 (18)
O1	0.083 (3)	0.123 (3)	0.0530 (18)	−0.064 (2)	−0.0270 (17)	0.0274 (19)
O2	0.0473 (17)	0.0572 (17)	0.0486 (16)	−0.0220 (14)	−0.0046 (13)	−0.0042 (13)
O3	0.069 (2)	0.088 (3)	0.121 (3)	−0.028 (2)	0.000 (2)	−0.057 (2)
N8	0.066 (3)	0.092 (3)	0.078 (3)	−0.029 (3)	−0.013 (3)	−0.013 (3)
O4	0.056 (3)	0.201 (6)	0.117 (4)	−0.030 (3)	−0.038 (3)	−0.021 (3)
O5	0.134 (5)	0.359 (11)	0.085 (4)	−0.056 (6)	−0.018 (3)	−0.084 (5)
O6	0.067 (3)	0.093 (3)	0.182 (5)	−0.010 (2)	0.017 (3)	−0.001 (3)
O7	0.0550 (19)	0.098 (3)	0.0602 (18)	−0.0392 (19)	−0.0137 (15)	−0.0114 (17)
O8	0.095 (3)	0.058 (2)	0.091 (2)	−0.0182 (19)	−0.057 (2)	0.0015 (17)
O9	0.089 (3)	0.063 (2)	0.079 (2)	−0.032 (2)	−0.0155 (19)	0.0027 (17)
O10	0.078 (3)	0.093 (3)	0.095 (3)	−0.039 (2)	−0.022 (2)	−0.006 (2)

### Geometric parameters (Å, °)

**Table:**

Ni1—N1	2.080 (3)	C17—C18	1.353 (6)
Ni1—N5	2.083 (3)	C17—H17	0.9500
Ni1—N6	2.087 (3)	C18—C19	1.428 (5)
Ni1—N3	2.088 (3)	C18—H18	0.9500
Ni1—N4	2.090 (3)	C19—C24	1.408 (5)
Ni1—N2	2.103 (2)	C19—C20	1.412 (5)
N1—C1	1.328 (4)	C20—C21	1.360 (6)
N1—C11	1.358 (4)	C20—H20	0.9500
N2—C10	1.326 (4)	C21—C22	1.396 (5)
N2—C12	1.369 (4)	C21—H21	0.9500
N3—C13	1.332 (4)	C22—H22	0.9500
N3—C23	1.356 (4)	C23—C24	1.435 (5)
N4—C22	1.330 (4)	C25—C26	1.407 (5)
N4—C24	1.359 (4)	C25—H25	0.9500
N5—C25	1.329 (4)	C26—C27	1.356 (5)
N5—C35	1.356 (4)	C26—H26	0.9500
N6—C34	1.325 (4)	C27—C28	1.402 (5)
N6—C36	1.361 (4)	C27—H27	0.9500
C1—C2	1.402 (6)	C28—C35	1.407 (4)
C1—H1	0.9500	C28—C29	1.434 (5)
C2—C3	1.367 (6)	C29—C30	1.350 (5)
C2—H2	0.9500	C29—H29	0.9500
C3—C4	1.402 (5)	C30—C31	1.429 (5)
C3—H3	0.9500	C30—H30	0.9500
C4—C11	1.400 (5)	C31—C36	1.399 (4)
C4—C5	1.436 (5)	C31—C32	1.410 (5)
C5—C6	1.346 (5)	C32—C33	1.363 (5)
C5—H5	0.9500	C32—H32	0.9500
C6—C7	1.420 (5)	C33—C34	1.397 (5)
C6—H6	0.9500	C33—H33	0.9500
C7—C12	1.407 (4)	C34—H34	0.9500
C7—C8	1.417 (5)	C35—C36	1.434 (5)
C8—C9	1.354 (5)	N7—O1	1.221 (5)
C8—H8	0.9500	N7—O3	1.245 (5)
C9—C10	1.399 (5)	N7—O2	1.265 (4)
C9—H9	0.9500	N8—O4	1.179 (6)
C10—H10	0.9500	N8—O5	1.199 (6)
C11—C12	1.443 (5)	N8—O6	1.266 (6)
C13—C14	1.388 (5)	O7—H71	0.8200
C13—H13	0.9500	O7—H72	0.8200
C14—C15	1.358 (6)	O8—H81	0.8200
C14—H14	0.9500	O8—H82	0.8200
C15—C16	1.403 (5)	O9—H91	0.8248
C15—H15	0.9500	O9—H92	0.8200
C16—C23	1.407 (5)	O10—H101	0.8200
C16—C17	1.437 (6)	O10—H102	0.8200
			
N1—Ni1—N5	95.20 (10)	C14—C15—H15	120.1
N1—Ni1—N6	92.06 (11)	C16—C15—H15	120.1
N5—Ni1—N6	79.80 (10)	C15—C16—C23	116.8 (4)
N1—Ni1—N3	96.51 (11)	C15—C16—C17	124.0 (3)
N5—Ni1—N3	93.96 (10)	C23—C16—C17	119.2 (3)
N6—Ni1—N3	169.85 (10)	C18—C17—C16	121.0 (4)
N1—Ni1—N4	170.85 (10)	C18—C17—H17	119.5
N5—Ni1—N4	93.40 (10)	C16—C17—H17	119.5
N6—Ni1—N4	92.55 (11)	C17—C18—C19	121.0 (4)
N3—Ni1—N4	79.75 (11)	C17—C18—H18	119.5
N1—Ni1—N2	79.88 (10)	C19—C18—H18	119.5
N5—Ni1—N2	172.77 (11)	C24—C19—C20	116.8 (3)
N6—Ni1—N2	95.00 (10)	C24—C19—C18	119.5 (3)
N3—Ni1—N2	91.88 (10)	C20—C19—C18	123.7 (4)
N4—Ni1—N2	91.84 (10)	C21—C20—C19	119.7 (3)
C1—N1—C11	117.6 (3)	C21—C20—H20	120.1
C1—N1—Ni1	128.6 (2)	C19—C20—H20	120.1
C11—N1—Ni1	113.6 (2)	C20—C21—C22	119.7 (3)
C10—N2—C12	117.9 (3)	C20—C21—H21	120.2
C10—N2—Ni1	129.9 (2)	C22—C21—H21	120.2
C12—N2—Ni1	112.1 (2)	N4—C22—C21	122.7 (3)
C13—N3—C23	117.7 (3)	N4—C22—H22	118.6
C13—N3—Ni1	129.7 (3)	C21—C22—H22	118.6
C23—N3—Ni1	112.5 (2)	N3—C23—C16	123.1 (3)
C22—N4—C24	118.0 (3)	N3—C23—C24	117.2 (3)
C22—N4—Ni1	129.4 (3)	C16—C23—C24	119.7 (3)
C24—N4—Ni1	112.2 (2)	N4—C24—C19	123.0 (3)
C25—N5—C35	117.9 (3)	N4—C24—C23	117.3 (3)
C25—N5—Ni1	129.3 (2)	C19—C24—C23	119.7 (3)
C35—N5—Ni1	112.7 (2)	N5—C25—C26	122.9 (3)
C34—N6—C36	117.8 (3)	N5—C25—H25	118.6
C34—N6—Ni1	129.7 (2)	C26—C25—H25	118.6
C36—N6—Ni1	112.4 (2)	C27—C26—C25	119.1 (3)
N1—C1—C2	122.8 (3)	C27—C26—H26	120.5
N1—C1—H1	118.6	C25—C26—H26	120.5
C2—C1—H1	118.6	C26—C27—C28	119.9 (3)
C3—C2—C1	119.4 (4)	C26—C27—H27	120.1
C3—C2—H2	120.3	C28—C27—H27	120.1
C1—C2—H2	120.3	C27—C28—C35	117.4 (3)
C2—C3—C4	119.4 (4)	C27—C28—C29	124.0 (3)
C2—C3—H3	120.3	C35—C28—C29	118.6 (3)
C4—C3—H3	120.3	C30—C29—C28	121.2 (3)
C11—C4—C3	117.4 (3)	C30—C29—H29	119.4
C11—C4—C5	119.1 (3)	C28—C29—H29	119.4
C3—C4—C5	123.5 (3)	C29—C30—C31	121.2 (3)
C6—C5—C4	120.4 (3)	C29—C30—H30	119.4
C6—C5—H5	119.8	C31—C30—H30	119.4
C4—C5—H5	119.8	C36—C31—C32	117.3 (3)
C5—C6—C7	122.4 (3)	C36—C31—C30	119.1 (3)
C5—C6—H6	118.8	C32—C31—C30	123.6 (3)
C7—C6—H6	118.8	C33—C32—C31	119.3 (3)
C12—C7—C8	116.6 (3)	C33—C32—H32	120.3
C12—C7—C6	118.7 (3)	C31—C32—H32	120.3
C8—C7—C6	124.7 (3)	C32—C33—C34	119.6 (3)
C9—C8—C7	119.8 (3)	C32—C33—H33	120.2
C9—C8—H8	120.1	C34—C33—H33	120.2
C7—C8—H8	120.1	N6—C34—C33	122.9 (3)
C8—C9—C10	120.0 (3)	N6—C34—H34	118.5
C8—C9—H9	120.0	C33—C34—H34	118.5
C10—C9—H9	120.0	N5—C35—C28	122.8 (3)
N2—C10—C9	122.5 (3)	N5—C35—C36	117.2 (3)
N2—C10—H10	118.7	C28—C35—C36	120.0 (3)
C9—C10—H10	118.7	N6—C36—C31	123.0 (3)
N1—C11—C4	123.3 (3)	N6—C36—C35	117.2 (3)
N1—C11—C12	116.8 (3)	C31—C36—C35	119.8 (3)
C4—C11—C12	119.9 (3)	O1—N7—O3	120.1 (4)
N2—C12—C7	123.1 (3)	O1—N7—O2	120.3 (4)
N2—C12—C11	117.4 (3)	O3—N7—O2	119.6 (4)
C7—C12—C11	119.5 (3)	O4—N8—O5	120.6 (6)
N3—C13—C14	122.8 (4)	O4—N8—O6	124.0 (6)
N3—C13—H13	118.6	O5—N8—O6	115.3 (6)
C14—C13—H13	118.6	H71—O7—H72	105.6
C15—C14—C13	119.7 (3)	H81—O8—H82	109.1
C15—C14—H14	120.2	H91—O9—H92	106.6
C13—C14—H14	120.2	H101—O10—H102	107.6
C14—C15—C16	119.9 (3)		
			
N5—Ni1—N1—C1	−4.6 (3)	C6—C7—C12—N2	−179.5 (3)
N6—Ni1—N1—C1	−84.5 (3)	C8—C7—C12—C11	−179.2 (3)
N3—Ni1—N1—C1	90.0 (3)	C6—C7—C12—C11	1.3 (5)
N2—Ni1—N1—C1	−179.2 (3)	N1—C11—C12—N2	−1.3 (4)
N5—Ni1—N1—C11	170.3 (2)	C4—C11—C12—N2	179.2 (3)
N6—Ni1—N1—C11	90.3 (2)	N1—C11—C12—C7	178.0 (3)
N3—Ni1—N1—C11	−95.2 (2)	C4—C11—C12—C7	−1.5 (5)
N2—Ni1—N1—C11	−4.4 (2)	C23—N3—C13—C14	−0.6 (5)
N1—Ni1—N2—C10	−178.5 (3)	Ni1—N3—C13—C14	175.1 (2)
N6—Ni1—N2—C10	90.3 (3)	N3—C13—C14—C15	−1.5 (5)
N3—Ni1—N2—C10	−82.2 (3)	C13—C14—C15—C16	1.9 (5)
N4—Ni1—N2—C10	−2.4 (3)	C14—C15—C16—C23	−0.3 (5)
N1—Ni1—N2—C12	3.7 (2)	C14—C15—C16—C17	179.1 (3)
N6—Ni1—N2—C12	−87.5 (2)	C15—C16—C17—C18	−178.7 (4)
N3—Ni1—N2—C12	99.9 (2)	C23—C16—C17—C18	0.7 (5)
N4—Ni1—N2—C12	179.7 (2)	C16—C17—C18—C19	1.0 (6)
N1—Ni1—N3—C13	−12.4 (3)	C17—C18—C19—C24	−1.5 (5)
N5—Ni1—N3—C13	83.3 (3)	C17—C18—C19—C20	178.0 (4)
N6—Ni1—N3—C13	134.9 (5)	C24—C19—C20—C21	0.4 (5)
N4—Ni1—N3—C13	176.0 (3)	C18—C19—C20—C21	−179.0 (3)
N2—Ni1—N3—C13	−92.5 (3)	C19—C20—C21—C22	−0.4 (5)
N1—Ni1—N3—C23	163.4 (2)	C24—N4—C22—C21	1.4 (5)
N5—Ni1—N3—C23	−100.9 (2)	Ni1—N4—C22—C21	−171.0 (2)
N6—Ni1—N3—C23	−49.3 (7)	C20—C21—C22—N4	−0.6 (5)
N4—Ni1—N3—C23	−8.1 (2)	C13—N3—C23—C16	2.2 (4)
N2—Ni1—N3—C23	83.4 (2)	Ni1—N3—C23—C16	−174.2 (2)
N5—Ni1—N4—C22	−85.2 (3)	C13—N3—C23—C24	−177.1 (3)
N6—Ni1—N4—C22	−5.2 (3)	Ni1—N3—C23—C24	6.5 (3)
N3—Ni1—N4—C22	−178.6 (3)	C15—C16—C23—N3	−1.8 (5)
N2—Ni1—N4—C22	89.8 (3)	C17—C16—C23—N3	178.8 (3)
N5—Ni1—N4—C24	102.1 (2)	C15—C16—C23—C24	177.6 (3)
N6—Ni1—N4—C24	−178.0 (2)	C17—C16—C23—C24	−1.9 (5)
N3—Ni1—N4—C24	8.7 (2)	C22—N4—C24—C19	−1.4 (4)
N2—Ni1—N4—C24	−82.9 (2)	Ni1—N4—C24—C19	172.3 (2)
N1—Ni1—N5—C25	85.3 (3)	C22—N4—C24—C23	178.3 (3)
N6—Ni1—N5—C25	176.5 (3)	Ni1—N4—C24—C23	−8.0 (3)
N3—Ni1—N5—C25	−11.6 (3)	C20—C19—C24—N4	0.5 (5)
N4—Ni1—N5—C25	−91.6 (3)	C18—C19—C24—N4	180.0 (3)
N1—Ni1—N5—C35	−99.1 (2)	C20—C19—C24—C23	−179.2 (3)
N6—Ni1—N5—C35	−7.9 (2)	C18—C19—C24—C23	0.3 (5)
N3—Ni1—N5—C35	164.0 (2)	N3—C23—C24—N4	1.1 (4)
N4—Ni1—N5—C35	84.1 (2)	C16—C23—C24—N4	−178.3 (3)
N1—Ni1—N6—C34	−82.2 (3)	N3—C23—C24—C19	−179.2 (3)
N5—Ni1—N6—C34	−177.1 (3)	C16—C23—C24—C19	1.4 (4)
N3—Ni1—N6—C34	130.3 (6)	C35—N5—C25—C26	−0.7 (5)
N4—Ni1—N6—C34	89.9 (3)	Ni1—N5—C25—C26	174.8 (3)
N2—Ni1—N6—C34	−2.2 (3)	N5—C25—C26—C27	−1.5 (6)
N1—Ni1—N6—C36	101.9 (2)	C25—C26—C27—C28	2.5 (5)
N5—Ni1—N6—C36	7.0 (2)	C26—C27—C28—C35	−1.4 (5)
N3—Ni1—N6—C36	−45.6 (7)	C26—C27—C28—C29	178.5 (3)
N4—Ni1—N6—C36	−86.0 (2)	C27—C28—C29—C30	−177.1 (3)
N2—Ni1—N6—C36	−178.1 (2)	C35—C28—C29—C30	2.8 (5)
C11—N1—C1—C2	−1.0 (5)	C28—C29—C30—C31	−0.2 (6)
Ni1—N1—C1—C2	173.7 (3)	C29—C30—C31—C36	−2.3 (5)
N1—C1—C2—C3	1.6 (6)	C29—C30—C31—C32	177.7 (3)
C1—C2—C3—C4	−0.6 (6)	C36—C31—C32—C33	−1.0 (5)
C2—C3—C4—C11	−0.9 (5)	C30—C31—C32—C33	178.9 (3)
C2—C3—C4—C5	179.4 (4)	C31—C32—C33—C34	−0.6 (6)
C11—C4—C5—C6	0.1 (5)	C36—N6—C34—C33	−0.1 (5)
C3—C4—C5—C6	179.9 (4)	Ni1—N6—C34—C33	−175.8 (3)
C4—C5—C6—C7	−0.3 (6)	C32—C33—C34—N6	1.2 (6)
C5—C6—C7—C12	−0.3 (5)	C25—N5—C35—C28	1.8 (5)
C5—C6—C7—C8	−179.9 (3)	Ni1—N5—C35—C28	−174.4 (2)
C12—C7—C8—C9	0.2 (5)	C25—N5—C35—C36	−176.1 (3)
C6—C7—C8—C9	179.7 (3)	Ni1—N5—C35—C36	7.7 (4)
C7—C8—C9—C10	−0.5 (5)	C27—C28—C35—N5	−0.8 (5)
C12—N2—C10—C9	−0.5 (5)	C29—C28—C35—N5	179.3 (3)
Ni1—N2—C10—C9	−178.2 (2)	C27—C28—C35—C36	177.1 (3)
C8—C9—C10—N2	0.7 (5)	C29—C28—C35—C36	−2.8 (5)
C1—N1—C11—C4	−0.6 (5)	C34—N6—C36—C31	−1.6 (5)
Ni1—N1—C11—C4	−176.1 (2)	Ni1—N6—C36—C31	174.8 (2)
C1—N1—C11—C12	179.9 (3)	C34—N6—C36—C35	178.4 (3)
Ni1—N1—C11—C12	4.4 (4)	Ni1—N6—C36—C35	−5.2 (4)
C3—C4—C11—N1	1.6 (5)	C32—C31—C36—N6	2.2 (5)
C5—C4—C11—N1	−178.7 (3)	C30—C31—C36—N6	−177.7 (3)
C3—C4—C11—C12	−179.0 (3)	C32—C31—C36—C35	−177.8 (3)
C5—C4—C11—C12	0.8 (5)	C30—C31—C36—C35	2.2 (5)
C10—N2—C12—C7	0.1 (5)	N5—C35—C36—N6	−1.7 (4)
Ni1—N2—C12—C7	178.2 (2)	C28—C35—C36—N6	−179.7 (3)
C10—N2—C12—C11	179.4 (3)	N5—C35—C36—C31	178.3 (3)
Ni1—N2—C12—C11	−2.5 (3)	C28—C35—C36—C31	0.4 (5)
C8—C7—C12—N2	0.0 (5)		

### Hydrogen-bond geometry (Å, °)

**Table:**

*D*—H···*A*	*D*—H	H···*A*	*D*···*A*	*D*—H···*A*
O7—H72···O9^i^	0.82	1.97	2.768 (5)	164
O8—H81···O7^i^	0.82	1.94	2.749 (5)	172
O8—H82···O2^ii^	0.82	2.04	2.827 (4)	164
O9—H91···O4	0.82	2.07	2.845 (6)	155
O9—H92···O6^i^	0.82	2.00	2.817 (6)	175
O10—H101···O8	0.82	2.01	2.822 (5)	169
O10—H102···O9	0.82	2.20	2.900 (6)	143
C5—H5···O5^iii^	0.95	2.53	3.293 (7)	138
C15—H15···O2^iv^	0.95	2.53	3.265 (5)	134
C25—H25···O7^v^	0.95	2.52	3.300 (5)	140
C32—H32···O4	0.95	2.46	3.165 (6)	131
C34—H34···O1^ii^	0.95	2.37	3.180 (5)	143

Symmetry codes: (i) −*x*, −*y*+1, −*z*+1; (ii) −*x*+1, −*y*, −*z*+1; (iii) *x*, *y*, *z*+1; (iv) −*x*+2, −*y*, −*z*+1; (v) −*x*+1, −*y*+1, −*z*+1.
